# Photo-accelerated fast charging of lithium-ion batteries

**DOI:** 10.1038/s41467-019-12863-6

**Published:** 2019-10-30

**Authors:** Anna Lee, Márton Vörös, Wesley M. Dose, Jens Niklas, Oleg Poluektov, Richard D. Schaller, Hakim Iddir, Victor A. Maroni, Eungje Lee, Brian Ingram, Larry A. Curtiss, Christopher S. Johnson

**Affiliations:** 1 Chemical Sciences and Engineering Division, Argonne National Laboratory, Lemont, IL 60439 USA; 2 Material Sciences Division, Argonne National Laboratory, Lemont, IL 60439 USA; 3 Center for Nanoscale Materials, Nanoscience and Technology Division, Argonne National Laboratory, Lemont, IL 60439 USA

**Keywords:** Chemistry, Energy science and technology, Materials science

## Abstract

Due to their exceptional high energy density, lithium-ion batteries are of central importance in many modern electrical devices. A serious limitation, however, is the slow charging rate used to obtain the full capacity. Thus far, there have been no ways to increase the charging rate without losses in energy density and electrochemical performance. Here we show that the charging rate of a cathode can be dramatically increased via interaction with white light. We find that a direct exposure of light to an operating LiMn_2_O_4_ cathode during charging leads to a remarkable lowering of the battery charging time by a factor of two or more. This enhancement is enabled by the induction of a microsecond long-lived charge separated state, consisting of Mn^4+^ (hole) plus electron. This results in more oxidized metal centers and ejected lithium ions are created under light and with voltage bias. We anticipate that this discovery could pave the way to the development of new fast recharging battery technologies.

## Abstract (web summary)

Here the authors show that illumination of a lithium manganese oxide cathode can induce efficient charge-separation and electron transfer processes, thus giving rise to a new type of fast lithium-ion battery charging.

## Introduction

Lithium-ion batteries (LIBs) must be slow-charged in order to restore the full capacity (stored energy) of the battery, as well as to promote longer battery cycle life. Depending on the physicochemical properties of the composite electrodes, fast DC charging is in principle feasible when engineered active particle physical morphologies^1,2^, or low electrode loadings of nanostructured materials are used^3,4^. However, these approaches result in limited volumetric energy densities (or the amount of stored energy per volume of material) within the battery pack. A new paradigm in battery technology is required in order to overcome these obstacles leading to improved battery lifetime and requisite fast charging, particularly for battery-only electric vehicles and portable electronics.

Lithium-transition metal oxide materials such as LiMn_2_O_4_ (LMO) are widely employed as cathodes for energy-storage applications^5–9^. One such material is LiMn_2_O_4_ oxide (henceforth abbreviated as *LMO*) with the spinel structure^10^. LMO is a technologically relevant cathode for LIBs due to only 6 % volume change during lithium intercalation reaction^10^. The removal of one lithium atom per formula unit (*x* = 1 limit) yields the cubic λ-Mn_2_O_4_ phase^10–12^, as shown in Eq. 1).

1 $${\mathrm{LiMn}}_2{\mathrm{O}}_4 \to {\mathrm{Li}}_{(1 - x)}{\mathrm{Mn}}_{\mathrm{2}}{\mathrm{O}}_{\mathrm{4}} + {\mathrm{xLi}}^ + + {\mathrm{xe}}^ - \left( {0 < x < 1} \right)$$

During cycling both lithium cations and electrons must flow into and out of the material. For charging (moving to the rhs of Eq. 1), LMO is bulk oxidized, lithium leaves the material, and lithium cations are reduced with lithium metal deposition at the opposite anode (in this case lithium metal); the electrons travel in the external circuit with high average potential ~ 4.1 V *vs*. Li^+/o^. Mn oxidation state of LMO will change from Mn^3+^ to Mn^4+^ depending on the state of charge of the battery and the amount of Li in the material^10,13^. Stoichiometric LMO is a mixed valence compound with an average Mn oxidation state of +3.5; the electronic conductivity is mediated through polarons, and the bandgap is about 2–3 eV (Supplementary Fig. 1)^14,15^. As such, broadband white light (henceforth abbreviated light) should create electron-hole polaron pairs in LMO, which then could charge separate under potential bias.

We report here that illumination of a spinel-type LiMn_2_O_4_ cathode induces efficient charge-separation leading to fast lithium-ion battery charging. The discovery that exposure of LMO to light lowers charge transport resistance can lead to new fast recharging battery technologies for consumer applications and battery-only electric vehicles.

## Results

### Light-accepting battery design

**Fig. 1:**
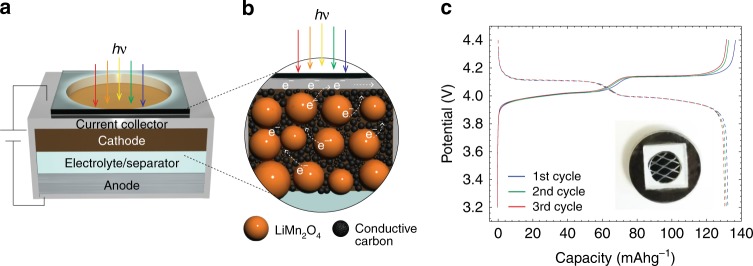
The principle of a photo-accelerated lithium-ion battery cell. The cell consists of **a** transparent window, current collector, cathode, electrolyte, separator, and anode. The broadband white light is used with an IR filter to avoid undesired heating of the cell. **b** Magnified view of the composite LMO cathode consisting of a Teflon binder, (e.g. –(CF_2_)_n_–), carbon particles as conductive diluent (e.g. acetylene carbon black), and active oxide powder. **c** Galvanostatic cycling voltage profile of a Li││1.2 M LiPF_6_; EC:EMC 3:7 (w:w)││LMO (EC = ethylene carbonate; EMC = ethyl methyl carbonate). Three cycles of charging (indicated in solid lines) and discharging (in dash lines) profiles between 3.2 and 4.4 V at a C/10 rate are shown. A photograph of a fabricated ‘open’ cell is shown in the inset

To probe the process, an ‘open’ light-accepting coin cell battery was developed. A schematic of our ‘open’ cell is illustrated in Fig. 1a wherein a transparent quartz window allows light to enter the cell. Figure 1b represents a magnified view of the LMO electrode with active particles, binder, and carbon conductive matrix. Galvanostatic cycling measurements confirm the operation of the ‘open’ cell (Fig. 1c), which is consistent with a conventional cell (Supplementary Fig. 2). The voltage profiles are as expected, whereby the reaction occurs over a voltage window of 3.2 to 4.4 V *vs*. Li^+/o^. The amount of capacity obtained is about 90% of the theoretical maximum, which is consistent with the literature^10,12,16^.

### Electrochemical performance

**Fig. 2:**
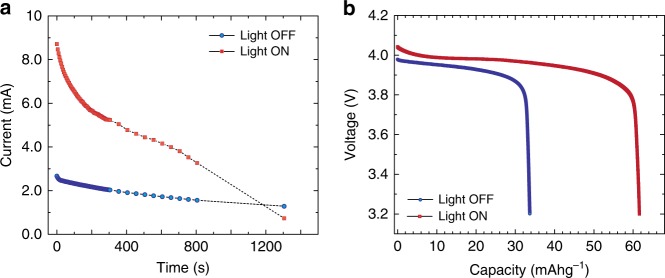
Electrochemical performance of the light accepting ‘open’ lithium-ion battery cell. Li││1.2 M LiPF_6_; EC:EMC 3:7 (w:w)││LMO (EC = ethylene carbonate; EMC = ethyl methyl carbonate). **a** Chronoamperometry curves for ‘light-on’ *vs*. ‘light-off’ state during constant voltage hold charging at 4.07 V (*vs*. Li^+/0^) for approximately 22 minutes. **b** Constant discharge curves carried out without illumination after the chronoamperometry experiment shown in **a** (voltage profiles at C/10). The “light-on” experiments were carried out under 1 SUN (100 mW/cm^2^), a white light is used with an IR filter to avoid undesired heating of the cell. The heat effect in control experiments are presented in Supplementary Fig. 10

In the electrochemical experiment, the ‘open’ cell is kept in either a ‘light-off’ state or a ‘light-on’ state, which is irradiated by white light from a Xe lamp fitted with an IR filter (see Methods). A direct-current (DC) experiment is carried out whereby a constant voltage of 4.07 V is applied to the ‘light-off’ state cell (blue curve in Fig. 2a), and the current is measured as a function of time. The applied voltage of 4.07 V (about 50% state-of-charge SOC) was chosen so as to provide a reasonable capacity from the battery cell, while also delivering a modest current over experimental time. As expected, a decay of current in time occurs as the reaction proceeds, removing Li cations from LMO and oxidizing Mn redox centers. The amount of charge from the cell is 2.21 C (or 26.1 mAh g^−1^ based on the oxide active weight = 23.5 mg). From the galvanostatic result in Fig. 1c, the amount of capacity expected at 4.07 V should be ~65 mAh g^−1^; clearly the ‘light-off’ state cell only reaches ~42% of this value. Indeed, even after 22 min the current is still 1.2 mA and the reaction continues. In contrast, integrating the charge passed over 22 min gives a capacity of 4.36 C (~52 mAh g^−1^) for the ‘light-on’ state (red curve in Fig. 2a), ~92% of the practical capacity.

The cell exhibits normal function after the light test and further, Raman spectroscopy of the LMO material suggests no phase changes, nor electrolyte decomposition/change (Supplementary Fig. 3). The UV/Vis absorption spectrum of the electrolyte was measured and was found to be transparent over the visible region of the spectrum, with minimal absorption in the UV portion. The spectral output of the Xe lamp used in the work has < 5% overall energy emitted in the UV region. With little light absorption across this spectral region, it is accurate to predict no overt degradation of the electrolyte under long term light exposure.

Galvanostatic (constant current) discharge without illumination (Fig. 2b) after the voltage hold experiments confirms that Li cations were de-intercalated from the LMO and the discharge capacity during Li re-insertion is consistent with the chronoamperometric (constant voltage charge) response in Fig. 2a. For this particular device, approximately twice the battery capacity is observed for the ‘light-on’ *vs*. ‘light-off’ condition. This corresponds to an increase of the charging rate by a factor of 3.4 compared to the ‘light-off’ state. Light enhancement has been reproduced in multiple devices with charging rate enhancement magnitudes ranging from 1.7 to 3.4 (Supplementary Fig. 4).

The current response delivered from 0 to 100% SOC (state-of-charge) was analyzed in potential step experiments in a sealed cell (Supplementary Fig. 5). Briefly the charge rate is near linearly flat over different SOC amounts up to 91% SOC. At the end of charge, about 10–100% higher currents are achieved between 91 and 99% SOC. At 99% SOC the charge rate decreases precipitously to below 10% of that from 0 to 44% SOC. This indicates that LMO is not limited in intrinsic charging rates at end of charge. Thus the light-induced photo-current provides the extra route to faster charging time in addition to the observed current driven potentiostatically.

### Transient absorption spectroscopy, electron paramagnetic resonance, and density functional theory calculations

**Fig. 3:**
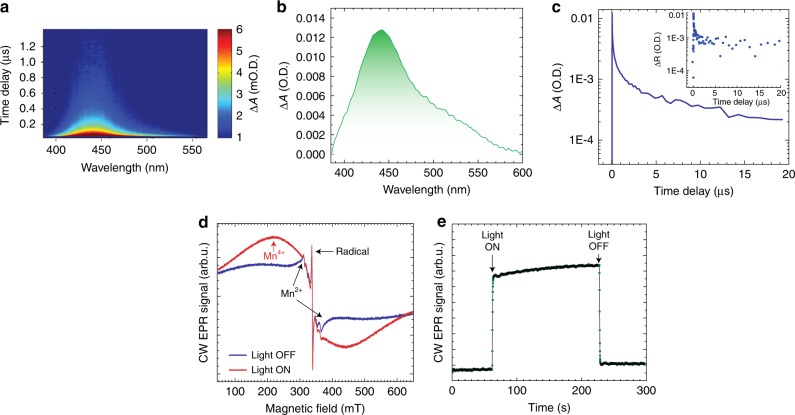
Probing the light induced processes through transient absorption (TA) and electron paramagnetic resonance (EPR) measurements. **a** A TA map, **b** spectrum at 0.026 µs pump-probe time delay, and **c** dynamics at 448 nm for an LMO film using 3.2 eV (390 nm) pump photons. Inset shows transient diffuse scattering experiments performed on a fully operational LMO composite cathode. **d** Continuous wave (CW) X-band EPR spectra of a charged LMO battery cathode in the ‘light-off’ state (i.e., before illumination shown in blue spectrum) and ‘light-on’ state (i.e., during illumination in red spectrum) with white light. T = 10 K. The CW EPR results in a derivative-type lineshape. e, Time dependence of the EPR signal at 210 mT before, during (‘light-on’), and after illumination (‘light-off’)

To probe mechanistic aspects of the light induced electrochemical processes in LiMn_2_O_4_, transient absorption (TA) spectroscopy, electron paramagnetic resonance (EPR), and density functional theory (DFT) calculations were investigated. The transient absorption map, spectra, and dynamics of a sputtered LMO thin-film only without other components in the composites (Supplementary Fig. 6) using 3.2 eV (390 nm) pump photons and a white light probe in a transmission geometry are shown in Fig. 3a–c, respectively. Optical excitation clearly results in a broad photo-induced absorption signal with a maximum near 2.8 eV (442 nm) extending down to 2.1 eV (590 nm). The induced absorption signal is consistent with generation of electron-hole pairs in the LMO film that can yield the modified charging rates in the operating illuminated cell. Importantly, the TA signal has long-lived components, with a trace that is characterized by decay components ~0.066, 2.5, and 10.3 μs. Indeed, fully operational LMO cathode composites show similar dynamic timescales in the transient diffuse reflectance (Fig. 3c inset). Similar to photovoltaic devices that require long lived excited states^17^, such long-lifetime may be beneficial for light-induced modification of a battery system.

To determine the chemical changes induced by optical excitation, EPR measurements were carried out with and without illumination at 10 K on a LMO electrode that had been potentiostatically charged at 4.07 V, then extracted from the cell. Without illumination (‘light-off’ state), a broad signal with a line width of several hundred mT was observed, centered at g ≈ 2 (≈0.34 T) corresponding to paramagnetic Mn^4+^ ions interacting with many close-by paramagnetic ions, like other Mn^4+^ or Mn^3+^ ions (Fig. 3d)^18,19^. Note that trivalent Mn^3+^ ions themselves are not directly observable under the experimental conditions. For the ‘light-off’ state, a narrow and intense signal centered at 340 mT (corresponding to *g* ≈ 2) is observed that we attribute to a radical species in the carbon/binder part of the composite cathode. The six-line signal with ~50 mT total width corresponds to Mn^2+^ ions^20–23^. The build-up of a separate Mn^2+^ phase has been reported for Li extraction from LiMn_2_O_4_ spinel^24^. Since Mn^2+^, even in small concentration, gives intense signals, the actual amount of Mn^2+^ impurities in the battery material may be relatively small. According to the spin quantification of the EPR spectra, the relative concentration of the Mn^2+^ ions is at least 3 orders of magnitude less than concentration of Mn^4+^ ions. Upon illumination (‘light-on’ state), a new distinctive broad signal with a width of ~215 mT (narrower than the broad ‘light-off’ Mn^4+^ signal) is created. This type of signal is typical for Mn^4+^ in LMO, but has different coordination and/or magnetic surrounding than the ‘light-off’ Mn^4+^ signal^18,19^. Note, that the intensity of the Mn^2+^ signal does not change upon illumination. Figure 3e shows that the signal corresponding to excess Mn^4+^ is created rapidly after the illumination starts, then it plateaus, and finally it drops close to its ‘light-off’ state after illumination is turned off. More importantly, the growth in the number of Mn^4+^ ions upon illumination indicates that Mn^3+^ is oxidized to Mn^4+^ (see Methods and Supplementary Figs. 7 and 8 on the effect of a control system).

## Discussion

From our experimental results, we propose a mechanism by which light assists fast charging of LMO. Upon illumination, photoexcited Mn^3+^ ([Mn^3+^]*) leads to the formation of Mn^4+^ (hole) and electron (Eq. 2). Specifically, under potential bias the electron percolates through the structure via charge transfer/polaron hopping towards the current collector, where the electron passes into the external circuit. This process is facilitated by the presence of the conductive carbon electrode network. In essence, from the photochemical process more Mn^3+^ is being oxidized to Mn^4+^ and subsequently more Li^+^ ions are ejected from the structure (i.e. the faster charging) than in the dark state. EPR indeed shows that, under photochemical conditions, the amount of oxidized Mn^4+^ species is found to be greater with “light-on” than “light-off” (Fig. 3d, e).

2 $${\mathrm{Mn}}^{3 + }\mathop{\longrightarrow}\limits^{{{\mathrm{h}}v}}\left[ {{\mathrm{Mn}}^{3 + }} \right]^ \ast \approx {\mathrm{Mn}}^{4 + }\left( {{\mathrm{hole}}} \right) + e^{-}\left( {{\mathrm{electron}}} \right)$$

3 $${\mathrm{2Mn}}^{3 + }\mathop{\longrightarrow}\limits^{{{\mathrm{h}}v}}2\left[ {{\mathrm{Mn}}^{3 + }} \right]^ \ast \approx {\mathrm{Mn}}^{4 + } + {\mathrm{Mn}}^{2 + }$$

In a parallel process, disproportionation reaction (Eq. 3) could also be a possibility occurring at the particle surface forming Mn^4+^ and Mn^2+^. Disproportionation reactions are calculated to occur at a surface of LMO enriching Mn^3+^ sites at an electrochemical surface environment^25^. Since light irradiation is shown (EPR) to produce Mn^4+^ (Mn^3+^ photo-oxidation), on static Mn^3+^ sites, without any further effect on the distribution and ordering of the rest of the Mn ions in the material. This process will in turn increase the local (surface) concentration of Mn^4+^ and reduce Mn^3+^, thereby reducing even further the possibility of Mn^3+^ disproportionation reaction that inherently requires a higher concentration of Mn^3+^. In fact DFT calculations show that disproportionation of Mn^3+^ on the surface would occur only for very specific configurations (local distribution of Mn^3+^ and Mn^4+^). Hence, we do not expect EPR to detect any Mn^2+^ in the static mode (no charging), or in the right-hand side of Eq. 3.

**Fig. 4:**
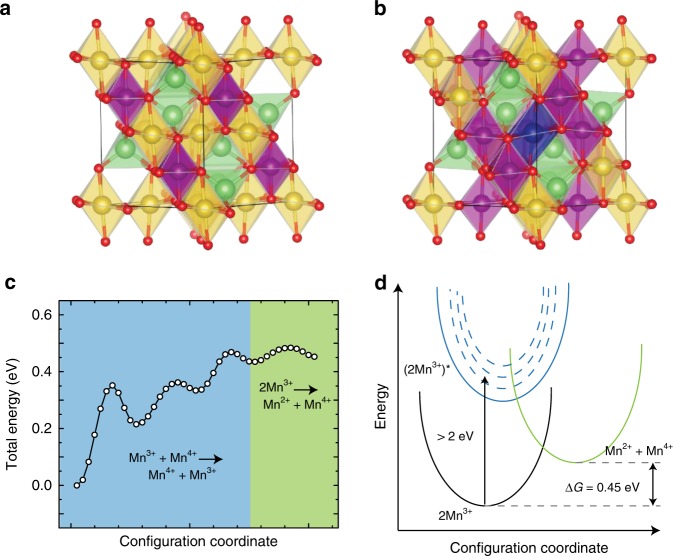
Atomistic studies of the proposed photo-induced charge separation mechanism in LMO. **a**, **b** Structural model of bulk stoichiometric LMO without and with disproportionation, respectively. Green, red, purple, yellow, blue spheres represent Li, O, Mn^4+^, Mn^3+^, and Mn^2+^ sites, respectively. **c** Relative ground state total energy along the transition path from the ground state to the disproportionated state. The first transition is a concerted electron exchange between two Mn^3+^ and Mn^4+^ sites, the second and third transition are single electron exchange processes between Mn^3+^ and Mn^4+^, and the last transition is the disproportionation reaction. **d** Schematic of the Eq. 3 scenario where photo-excited electron transfer process occurs from the ground state, through the excited states (2[Mn^3+^]*) to the disproportionated state (2Mn^3+^ → Mn^2+^ + Mn^4+^)

Instead, under voltage hold bias, the current flows via a polaron conduction process, where Mn^4+^ and Mn^3+^ continuously exchange charge, and creating new arrangements and ordering of (Mn^3+^-Mn^4+^) patterns, increasing the electronic entropy of the material. The shuffling between the Mn^4+^ and Mn^3+^ allows for a continuous and an increase sampling of new configurations that will be favorable for Mn^3+^ disproportionation reaction. The energy provided by light absorption will further help the reaction to occur (last step in green shaded region in Fig. 4c DFT energy profile). Note that the broad band of light will provide absorption even at lower wavelengths than required for photo-oxidation. Absorption at low energies can be enough to help disproportionation, which further increases the carrier density.

Note that delithiation (charge) is a process in which an electron and a Li^+^ are removed from the material at the same time. DFT results show that the energy required to remove a Li^+^ from the material is reduced near higher concentrations of Mn^4+^ (allowing for fast charging), but also even more reduced near Mn^2+^ (product of Mn^3+^ disproportionation). This later result, suggest a very short lifetime for Mn^2+^ during charging.

Overall, both photo-oxidation and disproportionation alone allow for fast charging, however, the combination of both processes is likely to be more efficient, as the applied bias will continuously feed and replenish the surface with fresh Mn^3+^ sites that will undergo photo-oxidation and disproportionation.

To support the disproportionation part of the mechanism, we performed density functional theory (DFT) calculations. Figure 4 shows the structural model of bulk LMO with and without disproportionation and the transition path between the ground state and disproportionated state (The details for model optimizations are in Supplementary information and Methods). The ground state structure of bulk LMO contains alternating and ordered Mn^3+^ and Mn^4+^ sites^15,26^. In order for disproportionation to happen this oxidation state pattern needs to be disordered by electron transfer processes as shown in the first steps of the transition path. The disproportionated structure is ~0.45 eV higher in energy than the ground state. The overall barrier energy of disproportionation is only about 0.5 eV, at least 1.5 eV lower than the optical gap of the system (~2 eV). This suggests that the light can provide the necessary excess energy to climb the calculated barrier. We find that Li extraction from the Mn^3+^/Mn^4+^ disordered or disproportionated LMO is energetically more favorable than extracting Li from Mn^3+^/Mn^4+^ ordered LMO without disproportionation (Supplementary Fig. 9). This might also have implications in build-up of Mn^3+^ at the electrode surface that can undergo disproportionation and follow-up electrochemical reaction more easily in the presence of light.

In summary, we have shown that light irradiation dramatically increases the charging rates of LMO in a conventional lithium-ion battery configuration under a potential bias. This is different than previous studies^27–29^ that combine photovoltaic and battery materials into one device to produce photoelectric conversion and storage efficiency and incorporating light-active photoelectrode materials, redox mediators, or dyes into batteries. Our work introduces a completely new route for dynamically altering the active material characteristics in lithium-ion batteries, in this case through light – matter interactions. While we have observed a significant reduction in charge time for our system, the overall technology improvement to realize faster recharge rates will need to establish engineering controls on the overall battery pack including the optimization of anode and electrolyte. We believe that this photo-accelerated charging process will open pathways towards rapid charging in future battery technologies.

## Methods

### LIB design and preparation

Cathode electrodes were prepared from a mixture of a Teflon binder, (e.g. –(CF_2_)_n_–), carbon particles as conductive diluent (e.g., acetylene carbon black), and active LiMn_2_O_4_ (NEI, Grade BE-30) powder in a weight ratio of 20:5:75 %, respectively and the assemblage was optimized. The binder keeps particles in laminate form, the carbon provides a conductive network amongst the particles, and active material oxide acts to support the electrochemical reaction and act as lithium cation host. After replenishing with isopropanol, the mixture was well-mixed and ground with a mortar and pestle until it formed a self-standing laminate. A thin sheet of laminate was achieved by manual rolling, after which it was dried at 75 °C in an oven and stored under vacuum. The free standing electrode was obtained by placing the pre-cut laminate (14 mm in diameter) onto an Al mesh (14 mm in diameter), then the laminate and the mesh were pressed together under hydraulic pressure (Carver Laboratory Equipment). The top of a 2032 coin cell was modified by punching a hole (8 mm in diameter) and then sealing the hole with Kapton tape prior to coin cell assembly. The modified coin cell was assembled with the cathode laminate, a glass fiber separator (Whatman® Glass microfiber filters, Grade GF/F), GenII electrolyte (1.2 M LiPF_6_ in 3:7 EC-EMC solution, Tomiyama Pure Chemical Industries), and a lithium metal anode (FMC Lithium) in an Argon-filled glove box. To probe an ‘open’ light-accepting coin cell battery, the tape was removed from the hole and a transparent quartz window (Technical glass products Inc.) was glued into place over the hole.

### Light and electrochemical experiments set up

For the ‘light’ experiments, a 300 W Xenon lamp (Atlas Specialty Lighting with a Perkin Elmer power supply) that spans from 300 nm to 1100 nm was used as a white light source and an IR filter (Newport) was used to avoid undesired heating of the cell. The spectral output of the Xe lamp used in the work has <5% overall energy emitted in the UV region. The incident light was normal to the sample and the temperature of the cell was measured by an IR thermometer focused on the transparent window. There was a temperature increase of ~7 °C when electrochemical measurements were carried out under ~1 SUN condition (100 mW cm^−2^). The heat effect in control experiments are presented in Supplementary Fig. 10. These results separate the heat contribution to the potentiostatic current due to thermal activation compared to photo-activation. Heating the ‘open’ cell at 35 °C (10 °C higher), increases the charge current by a factor 1.16 (not doubled), and at 45 °C, a factor of 1.26 was realized over that of 25 °C in “light-off” condition.

Cycle performance of the modified cells and of conventional (un-modified) cells were compared by measurements involving galvanostatic charge and discharge of the cells between 3.2 and 4.4 V at various C rates ranging from 2 C to C/10 (calculated based on a theoretical capacity of 148 mAh g^−1^ for LiMn_2_O_4_). The active LiMn_2_O_4_ material in an electrode typically weighed between 20 and 25 mg. Chronoamperometry was carried out by using a Gamry and Solartron SI1260 Frequency Response Analyzer. ‘Light-on’ and ‘light-off’ state chronoamperometry measurements were performed by holding a constant voltage of 4.07 V (vs. a Li metal counter electrode). All relevant data processing/analysis was done in Matlab (Mathworks Inc., Natick).

### Raman spectroscopy

Raman measurements were performed using a Renishaw inVia Raman microscope equipped with a 785 nm excitation laser. A 1200 l/mm grating was used in conjunction with a Ren 578 CCD detector. The laser power delivered to the sample surface was < 2 mW; the laser spot diameter was approximately 10 µm. Spectra were recorded through the window of the cell (BaF_2_ or quartz) using a 50-X objective with a numerical aperture of 0.5. A total of ten co-added 20 second exposures were made at each of the probed spots in the rectangular map. Other details of the Raman measurements and the spectra processing/analysis are the same as presented in refs. ^25,30–32^.

### Absorption and transient absorption measurements

Absorption measurements were carried out using a Cary 5000 UV-Vis-NIR spectrophotometer. The UV/Vis absorption spectrum of the electrolyte was measured and was found to be transparent over the visible region of the spectrum, with minimal absorption in the UV portion. With little light absorption across this spectral region, it is accurate to predict no overt degradation of the electrolyte under long term light exposure.

Spectrally-resolved transient absorption (TA) measurements were performed using a Helios EOS spectrometer and an amplified Ti:sapphire laser operating at 1 kHz that was tuned to produce 390 nm pump pulses using an optical parametric amplifier. White light probe pulses were derived from a picosecond Nd:YAG laser and photonic crystal fiber. A portion of the probe pulse was beamsplit to produce a reference laser shot for noise reduction. Pump-probe delay time was controlled and evaluated electronically. For TA measurements, two samples were used (a LMO only film as a control and LMO composite electrodes used in fully operational ‘open’ cells). For the LMO film preparation, a ~ 200 nm film of LMO was deposited on a quartz substrate (Technical Glass Products, USA) with a commercial sputtering system (AJA International, USA). Deposition was conducted by RF magnetron sputtering of a stoichiometric LMO target (>90% density) in argon at 3.1 mTorr and room temperature. The net RF power was 75 W and the deposition rate was ~6 Å min^−1^. A lithium overpressure was provided by DC sputtering from a Li-metal target at 15 W set off axis by 20°. The deposition times were 9 hours for ~300–350 nm films. The as-deposited LMO films were found to be amorphous; however a subsequent anneal air at 800 °C for 16 h resulted in a crystalline/polycrystalline films.

### Electron paramagnetic resonance (EPR) experiments

Continuous wave (CW) X-band (9–10 GHz) EPR experiments were carried out with a Bruker ELEXSYS II E500 EPR spectrometer (Bruker Biospin, Rheinstetten, Germany), equipped with a TE_102_ rectangular EPR resonator (Bruker ER 4102st). A helium gas-flow cryostat (ICE Oxford, UK) and an ITC503 from Oxford Instruments, UK, were used for measurements at cryogenic temperatures (T = 10 K). Light excitation was done directly in the resonator with a 300 W Xenon lamp (LX 300 F from Atlas Specialty Lighting with PS300–13 300 W power supply from Perkin Elmer). A water filter was used in combination with a KG2 short pass filter (Schott) to avoid unwanted heating of the sample. A GG400 long pass filter (Schott) was used to remove UV light. Data processing was done using Xepr (Bruker BioSpin, Rheinstetten) and Matlab 7.11.1 (The MathWorks, Inc., Natick) software. Simulations were performed using the EasySpin software package (version 5.0.20).

### Density functional theory (DFT) calculations

We carried out our atomistic calculations using the plane-wave density functional theory (DFT) code Quantum-Espresso^33^. Electron-nuclei interaction was taken into account by using recently developed ONCV norm-conserving pseudopotentials^34–36^. The wave function energy cutoff was 80 Ry. We used a unit cell with formula Li_8_Mn_16_O_32_ and 4 × 4 × 4 k-point sampling. The cell was optimized at the PBE + U level of theory with a U_Mn_ = 3.5. Previous studies showed that taking into account Jahn-Teller distortion and using hybrid or DFT + U levels of theory are essential for describing the charge localization that accompanies the two different oxidation states of Mn^15,37^. On average, the oxidation state of Mn is 3.5, which turns to formally 3 and 4, when the cubic unit cell is Jahn–Teller distorted into a tetragonal structure. It was also demonstrated that ferromagnetic (FM) and antiferromagnetic (AFM) arrangements of Mn ions are very close in energy, although the AFM ordering was shown to be slightly more stable^15^. More importantly, the optical properties were similar with AFM and FM ordering, we thus restricted our studies to FM ordering to avoid the complications arising from the multiple possible magnetic solutions when searching for the disproportionated state. To ensure the robustness of the results, we also performed PBE0 hybrid functional calculations. It was shown for many semiconductors that the mixing fraction entering the PBE0 hybrid functional should be chosen as the inverse of the high frequency dielectric constant. For LMO, the dielectric constant is 4.78 at the HSE06 level of theory^15^, suggesting that the original PBE0 mixing fraction of 0.25 might give rise to reliable quasiparticle band gaps. PBE0 calculations were carried out at the DFT + U geometry with a Monkhorst-Pack (MP) grid of 2 × 2 × 2, a momentum transfer grid of 1 × 1 × 1 and a reduced 80 Ry energy cutoff for the Fock operator. For the PBE0 calculations, we used a locally modified version of Quantum-Espresso to start PBE0 calculations with PBE + U wave functions. This not only allowed us to reach convergence much faster than from a PBE starting point, but it also made sure that the right magnetic state is reached. We found that the energy difference between the ground state and the disproportionated state was 0.72 eV at the PBE0 level of theory, which is in good qualitative agreement with that obtained with DFT + U (0.45 eV). See Supplementary Fig. 9 for a comparison of the DOS obtained at the DFT + U and PBE0 levels of theory.

We monitored oxidation states and local magnetizations using atomic projected charges and spin densities in QE and then verified the results by using a Maximally Localized Wannier Function (MLWF) approach. In particular, we computed MLWFs in QBOX using the same pseudopotentials that we used in QE^38^. We used the PBE0 hybrid functional, a reduced MP grid of the Gamma-point only and a wave function energy cutoff of 60 Ry. Since LMO is a strongly ionic system, the Wannier centers are located in close proximity to atoms. We thus counted the number of Wannier centers close to each atom and defined the oxidation state as the number of valence electrons minus the number of Wannier centers associated with that atom. The magnetic moment was computed by subtracting the number of up spin Wannier centers from the number of down spin Wannier centers. This approach of assigning oxidation states to atoms in solids is similar to what has been proposed in the literature by Jang et al.^39^, where the oxidation state was defined using the number of Wannier centers that move with an atom if the atom is displaced by a lattice vector^39,40^.

As described in the main text, the ground state structure of bulk LMO contains alternating and ordered Mn^3+^ and Mn^4+^ sites. We first constrained an excess electron on several different Mn^3+^ sites (making the chosen site Mn^2+^) with the excess electron being spontaneously withdrawn from another Mn^3+^ site (to become Mn^4+^). Having optimized the structure under this charge constraint, we further re-optimized the structure with the constraint released. We obtained the disproportionated structure by constraining the charge at a Mn site. We tried several different methods to constrain an excess charge: (i) increasing/decreasing the U of the selected Mn atom, (ii) charging the supercell, (iii) constraining the Mn-O bond lengths to ~2.2 Å (see Supplementary Fig. 10) for the Mn–O bond lengths in different Mn oxidation states). At the end, we found the disproportionated structure by a combination of (i) and (ii).

Although we performed our calculations on bulk LMO, we expect our results to qualitatively hold also for surfaces of LMO. To examine the effect of surfaces, we simulated a spherical stoichiometric cluster of LMO that has both high and low Miller index surfaces. The formula of the cluster was Li_14_Mn_28_O_56_ and the size was chosen so as to have bulk-like Mn atoms in the core. During the construction of the cluster we removed any Mn sites that had fewer than three Mn-O bonds. We found that under-coordinated Mn atoms (Mn atoms that have only three Mn-O bonds) naturally turn into Mn^2+^ without any energy barrier. Our result that disproportionation is more likely to happen on surfaces is further supported by the substantial experimental evidence that suggests that dissolution of Mn from the surface of LMO to the electrolyte is due to Mn disproportionation on the surface^41^. Furthermore, recent theoretical studies found that disproportionation of LMO might be an energetically favorable process on certain higher Miller index surfaces^42^.

## Supplementary information

Supplementary Information

## Data Availability

The data that support the findings of this study are available from the corresponding author upon request.

## Code availability

The computer code that support the findings of this study are available from the corresponding author upon request.
