# Supplementary material for: Photo-accelerated fast charging of lithium-ion batteries
Source: Nat Commun. 2019 Oct 30;10:4946. doi: 10.1038/s41467-019-12863-6 (PMC6821779; doi:10.1038/s41467-019-12863-6)
Supplement: Supplementary file 1 — Supplementary Information [file 41467_2019_12863_MOESM1_ESM.pdf]

Supplementary Information

Photo-Accelerated Fast Charging of Lithium-Ion Batteries

Lee et al.

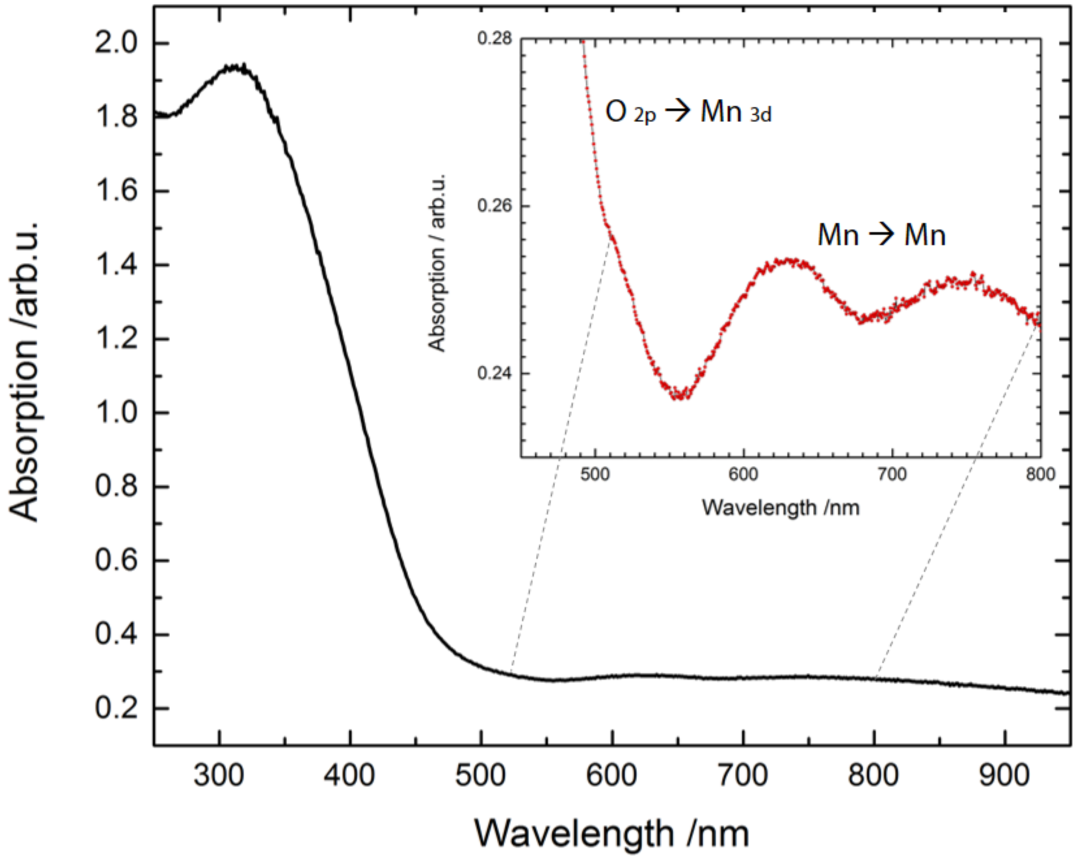

Supplementary Figure 1. Absorption spectrum of the $LiMn_2O_4$ film. The estimated bandgap is 2–3 eV. Inset highlights the expanded x-axis between 500 and 800 nm. The spectrum features a strong near UV-based electronic transition peaked at 310 nm which is ascribed to transitions from O 2p valence bands to Mn 3d conduction bands. The visible transitions at ~635 and 750 nm are weak and associated with Mn–Mn 3d transition.

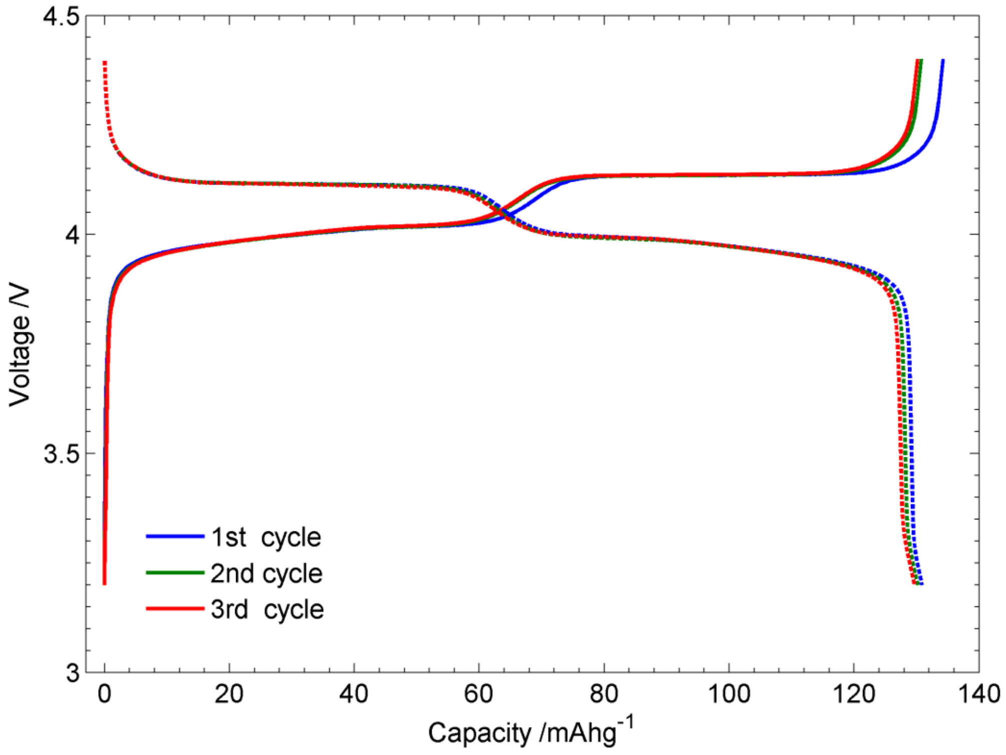

Supplementary Figure 2. Galvanostatic cycling voltage profile of a Li││1.2 M $LiPF_6$; EC:EMC 3:7 (w:w)││$LiMn_2O_4$ electrochemical 'closed' cell as a control system (EC = ethylene carbonate; EMC = ethyl methyl carbonate). Three cycles of charge (indicated in solid lines) and discharge (indicated in dash lines) are shown.

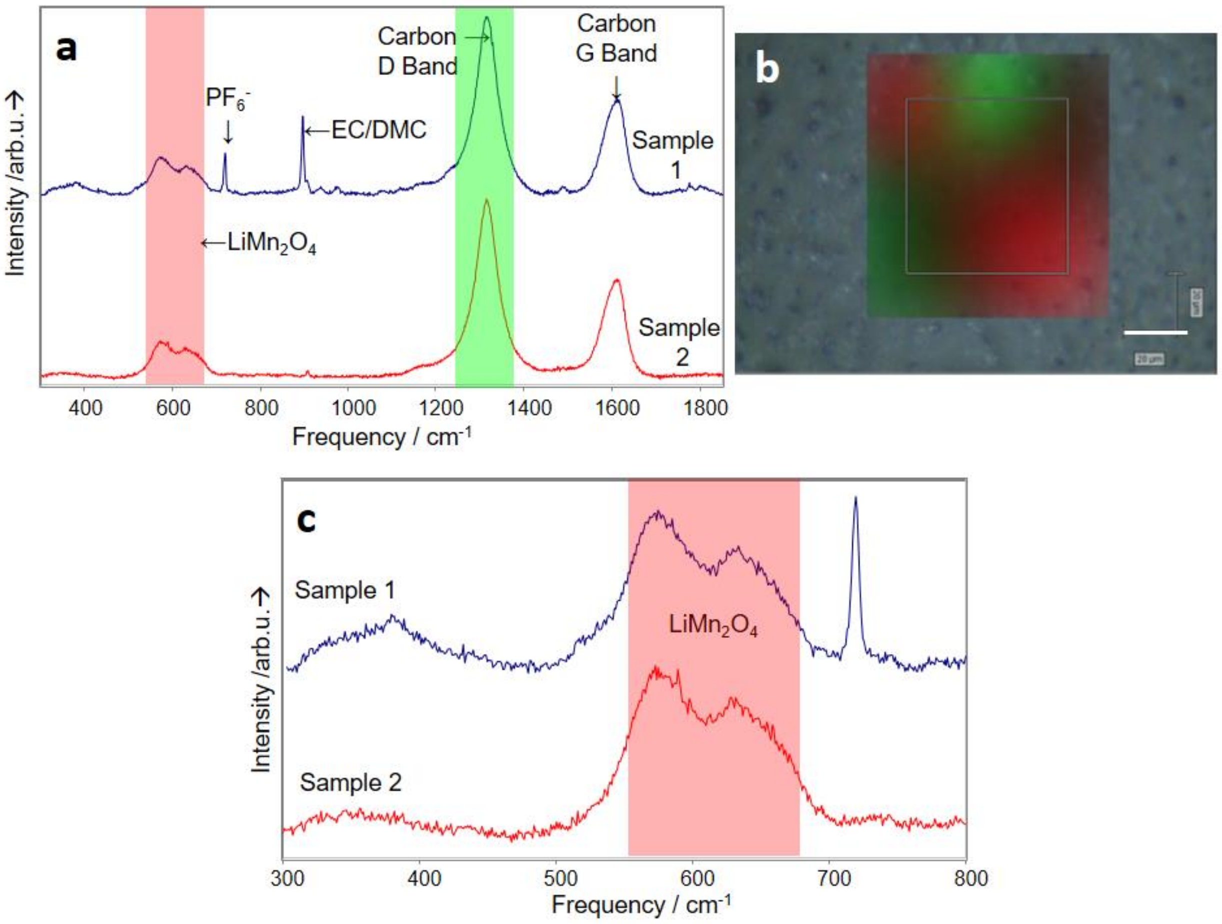

Supplementary Figure 3. Averaged in-situ Raman spectra for $LiMn_2O_4$ cathodes in half cell embodiments. The averaged spectrum (9 spots) for Sample 1 was taken prior to cycling, the averaged spectrum (16 spots) for Sample 2 was taken in the discharged state after cycling as described in the text. **(a)** shows the full spectral range scanned for each sample. **(b)** A chemical map of a portion of the Sample 2 cathode surface in the area where the multi-spot spectra were recorded is shown. The green shaded areas indicate where the carbon spectrum was relatively more intense; the red shaded areas indicate where the $LiMn_2O_4$ spectrum was relatively more intense. Scale bar is 20 µm. (c) An expanded view of the spectral region where the lattice vibrations of the $LiMn_2O_4$ phase appear is provided. These Raman results are generally consistent with previously published findings for $Li_{1+\delta}Mn_{2-\delta}O_4$.[1-4] The spectra indicate that the

electrode film may be preferentially oriented (viz. the relative intensities of the 630 and 580 cm^{-1} bands). For example, the ca. 630 cm^{-1} eigenvector could be perpendicular to the substrate and the ca. 580 cm^{-1} eigenvector could be parallel to the substrate.

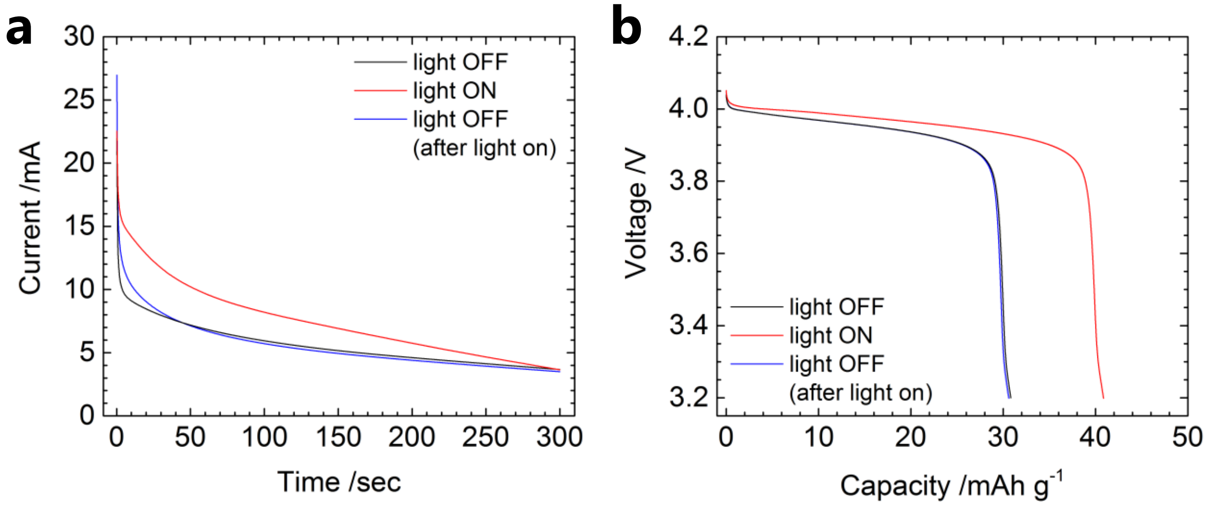

Supplementary Figure 4. Electrochemical performance of additional light accepting 'open' lithium ion battery cell. Li││1.2 M $LiPF_6$; EC:EMC 3:7 (w:w)││LMO (EC = ethylene carbonate; EMC = ethyl methyl carbonate). **(a)** Charge (chronoamperometry at 4.07 V vs. $Li^{+/o}$ for 5 minutes), and **(b)** discharge (galvanostatic discharge at C/10). Charge/discharge capacities in the 'light off' state yielded 30.14/29.92 mAh g^{-1} and 29.55/29.28 mAh g^{-1} before and after the 'light on' experiment respectively. In the 'light on' state the charge/discharge capacities were 41.60/40.87 mAh g^{-1}, representing a capacity increase of 1.38 times or an increase in the charging rate by a factor of 1.7 compared to the 'light-off' state.

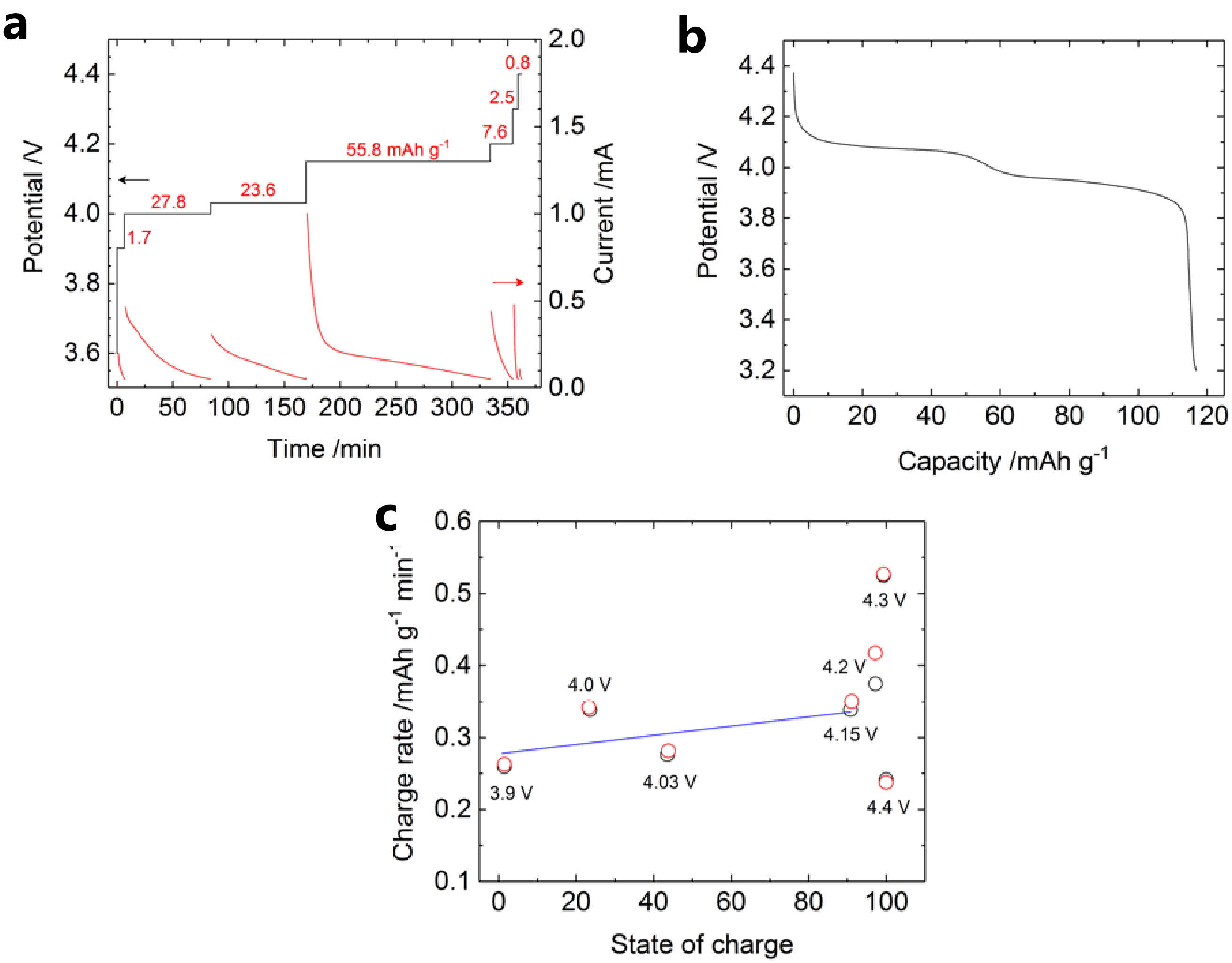

Supplementary Figure 5. Additional electrochemical measuremetns showing current response, potential profile, and charging rate. **(a)** Representative stepped electrochemical potential profile and current response during the third charge of $LiMn_2O_4$ versus Li metal in a sealed coin cell. The numbers in red indicate the capacity (in mAh g^{-1}) delivered at each potential step. The first and second charges were performed with the same protocol. **(b)** Representative electrochemical potential profile for the third discharge after stepped potential charge of $LiMn_2O_4$ versus Li metal at C/10 in a sealed coin cell. The first and second discharges were performed with the same protocol. **(c)** Charging rate (defined as the capacity delivered per unit time) with state of charge

during the third charge of $LiMn_2O_4$ versus Li metal in a sealed coin cell. Values for two cells are shown (black and red data). The potential corresponding to each state of charge is indicated. Blue line to guide the eye only.

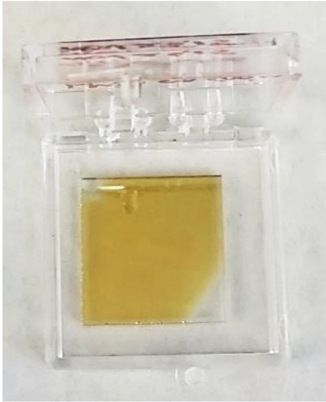

Supplementary Figure 6. A photograph of a thin-film (~200 nm) sputtered $LiMn_2O_4$ oxide on a quartz substrate (See Methods section for details).

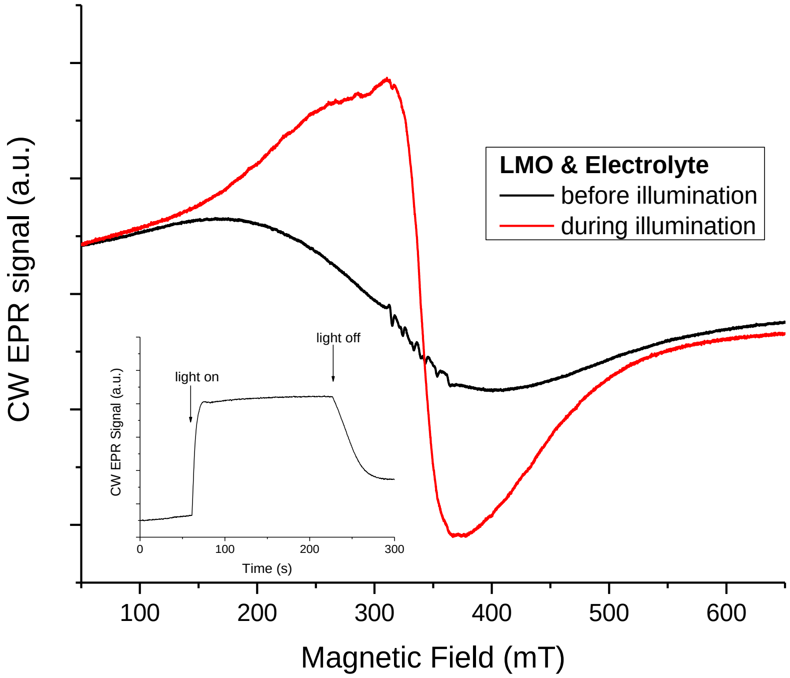

Supplementary Figure 7. Continuous wave (CW) X-band EPR spectra of a mixture of $LiMn_2O_4$ spinel and electrolyte before illumination (black spectrum) and during illumination (red spectrum) with a white light. T = 10 K. Inset shows time dependence of the EPR signal at 280 mT before, during, and after illumination. Note that the CW EPR results in a derivative-type line shape.

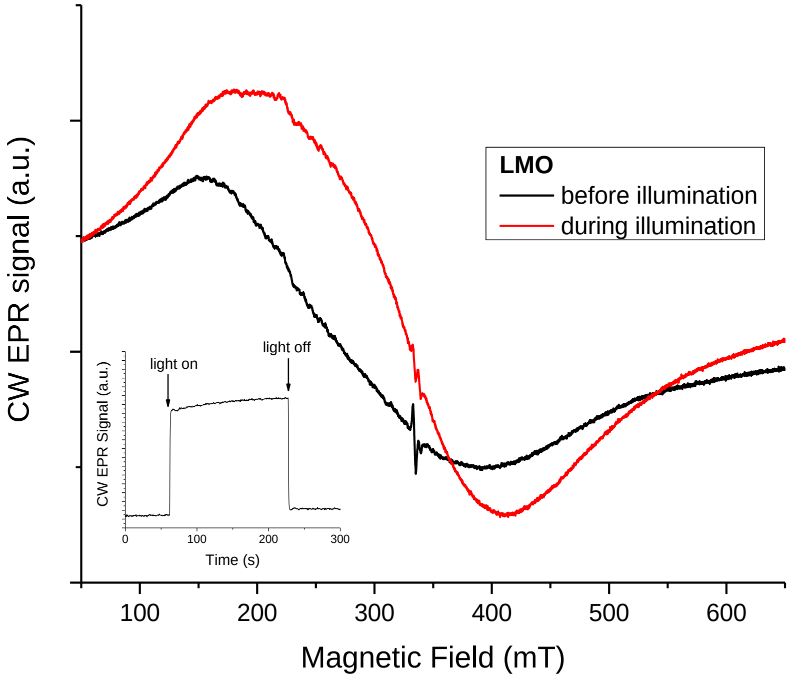

Supplementary Figure 8. Continuous wave (CW) X-band EPR spectra of $LiMn_2O_4$ spinel before illumination (black spectrum) and during illumination (red spectrum) with a white light. T = 10 K. Inset shows time dependence of the EPR signal at 280 mT before, during, and after illumination. Note that the CW EPR results in a derivative-type lineshape.

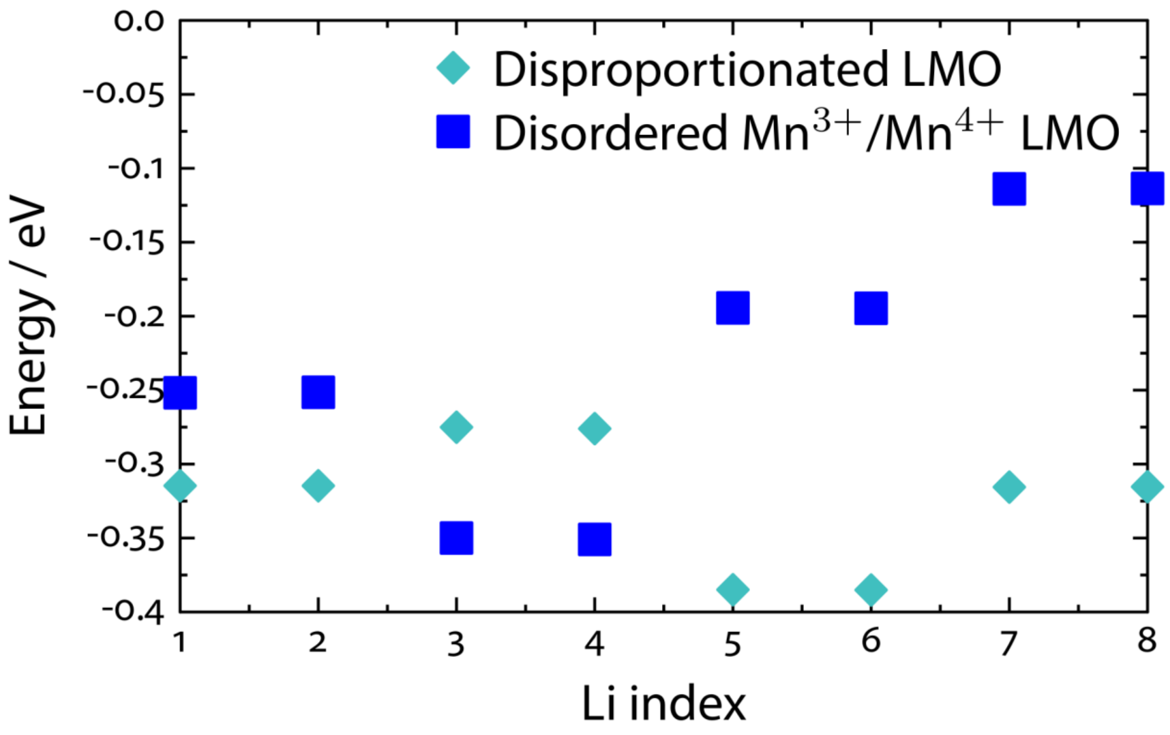

Supplementary Figure 9. Total energy differences of the first delithiation ($E(Li_7Mn_{16}O_{32})$-$E(Li_8Mn_{16}O_{32})$) for LMO with disproportionation and with Mn^{3+}/Mn^{4+} oxidation state disorder (the last local minimum before disproportionation in Fig. 4b) relative to the most stable ground state structure as a function of the index of the removed Li atom. Negative values show that it is easier to delithiate LMO with disproportionation or with Mn oxidation state disorder than perfect ordered LMO. The data also shows that disproportionated LMO is the easiest to delithiate. In the case of the disproportionated structure, we found that Li ($=Li^+ + e^-$) removal concomitantly oxidizes Mn^{2+} to Mn^{3+}. This is also in agreement with the fact that the highest occupied states are localized on Mn^{2+} (see Supplimentary Figure 11) and suggest that Mn^{2+} sites are the first to oxidize upon Li removal.

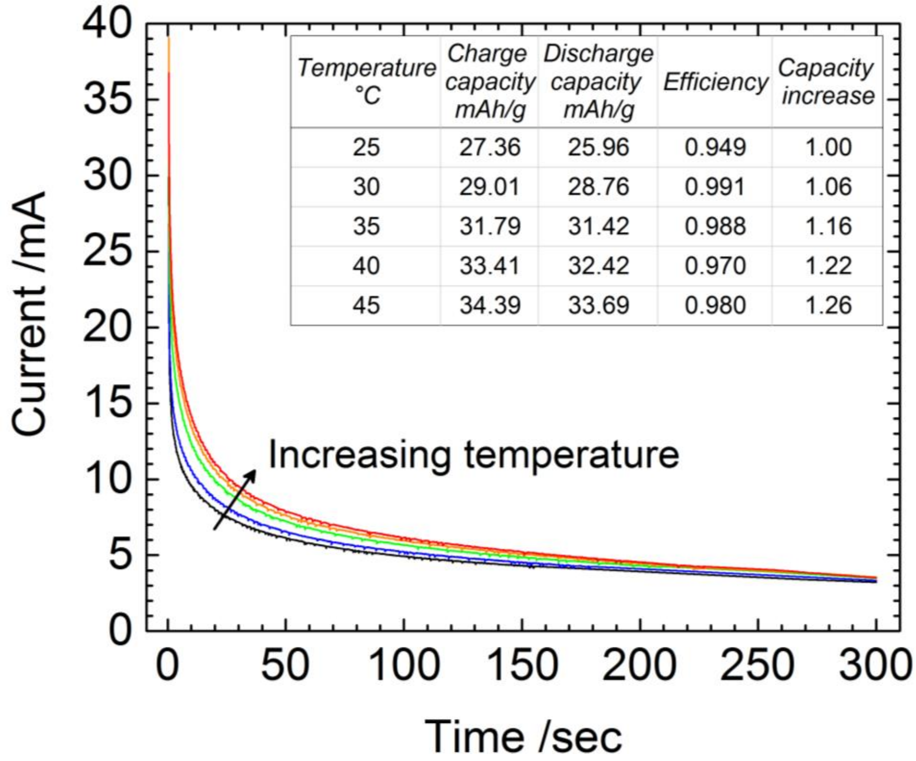

Temperature °C	Charge capacity mAh/g	Discharge capacity mAh/g	Efficiency	Capacity increase
25	27.36	25.96	0.949	1.00
30	29.01	28.76	0.991	1.06
35	31.79	31.42	0.988	1.16
40	33.41	32.42	0.970	1.22
45	34.39	33.69	0.980	1.26

Supplementary Figure 10. Electrochemical performance of a light accepting ‘open’ lithium ion battery cell during charge (chronoamperometry at 4.07 V vs. $Li^{+/o}$ for 5 minutes) with respect to temperature. Li││1.2 M $LiPF_6$; EC:EMC 3:7 (w:w)││LMO (EC = ethylene carbonate; EMC = ethyl methyl carbonate). The temperature was controlled to within ±0.2 °C using a Maccor heat/cool temperature chamber (MTC-010) and the cell was allowed to equilibrate at each temperature for 2 h before testing. The charge and discharge capacities, along with the cycling efficiency and capacity increase compared to the 25 °C experiment, are shown in the table inset. In the elevated temperature experiments the capacity increase ranged from 1.06 – 1.26, or the charging rate increased by a factor of 1.09 – 1.45, compared to the 25 °C experiment.

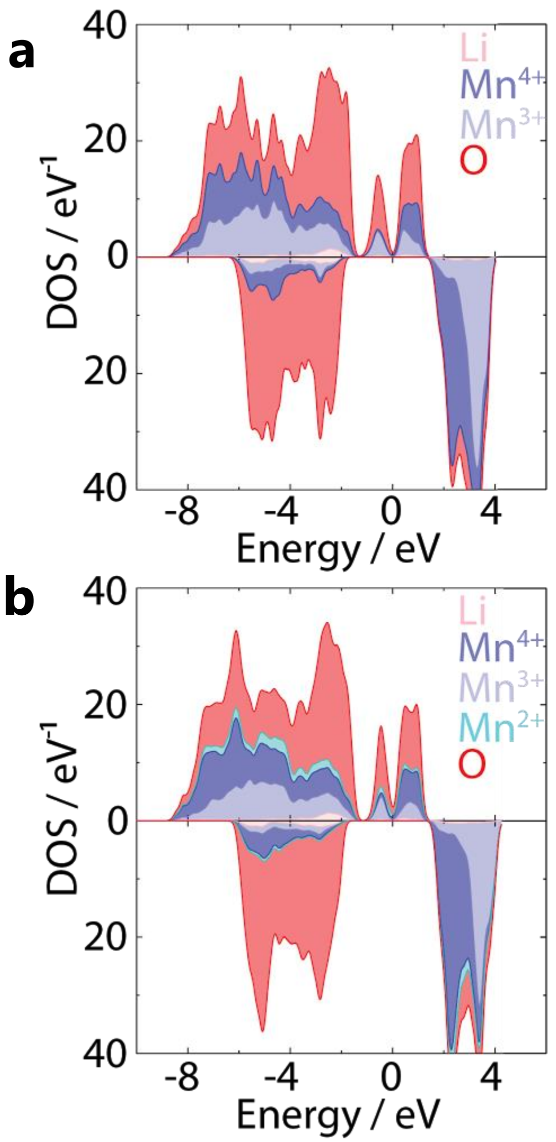

Supplementary Figure 11. Theoretical calculations. **(a)** Projected density of states (PDOS) of the ferromagnetic ground state of LMO using DFT+U(U_{Mn}=3.5) (left) and the PBE0 functional (right). **(b)** PDOS of the ferromagnetic ground state of disproportionated LMO using DFT+U (left) and the PBE0 functional (right). The hybridization between O and Mn states (the relative weight of O and Mn in the PDOS) is very similar at the DFT+U and PBE0 levels of theory. Interestingly, LMO is semimetallic at the DFT+U level of theory, but a gap of about 2 eV opens

at the PBE0 level of theory. Given the experimental evidence (see Supplimentry Figure 12 and Refs.[5,6]) that the optical gap should be as large as 2 eV and the fact that the PBE0 mixing fraction is close to what dielectric dependent hybrid functional suggests[7], we expect that the PBE0 electronic structure to be more accurate in terms of describing the band gap[8].

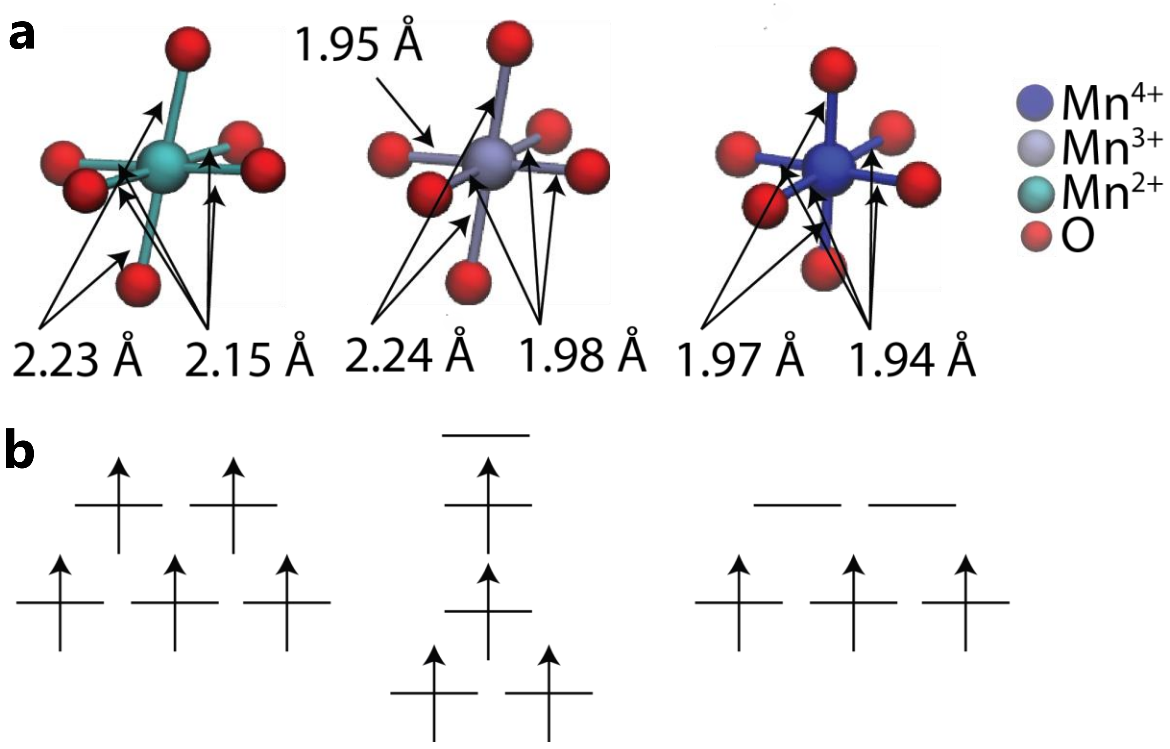

Supplementary Figure 12. Mn-O bond lengths in different Mn oxidation states. **(a)** Local geometries of Mn in different oxidation states, from left to right: Mn^{2+}, Mn^{3+}, Mn^{4+}. The Mn-O bond lengths are also shown. **(b)** Cartoon of the splitting of the Mn d states in high spin state for the respective oxidation states. Mn^{2+} and Mn^{4+} are in an almost octahedral environment, while the local symmetry of Mn^{3+} shows a tetragonal elongation of the octahedral environment (Jahn-Teller effect).

Supplementary References

1. Ramana, C. V., Massot, M. & Julien, C. M. XPS and Raman spectroscopic characterization of $LiMn_2O_4$ spinels. *Surf. Interface Anal.* **37**, 412–416 (2005).
2. Hwang, S.-J., Park, D.-H., Choy, J.-H. & Campet, G. Effect of chromium substitution on the lattice vibration of spinel lithium manganate: A new interpretation of the raman spectrum of $LiMn_2O_4$. *J. Phys. Chem. B* **108**, 12713–12717 (2004).
3. Chitra, S. *et al.* Characterization and electrochemical studies of $LiMn_2O_4$ cathode materials prepared by combustion method. *J. Electroceram.* **3**, 433–441 (1999).
4. Ammundsen, B., Burns, G. R., Islam, M. S., Kanoh, H. & Rozière, J. Lattice dynamics and vibrational spectra of lithium manganese oxides: A computer simulation and spectroscopic study. *J. Phys. Chem. B* **103**, 5175–5180 (1999).
5. Raja, M. W., Mahanty, S., Ghosh, P., Basu, R. N. & Maiti, H. S. Alanine-assisted low-temperature combustion synthesis of nanocrystalline $LiMn_2O_4$ for lithium-ion batteries. *Mater. Res. Bull.* **42**, 1499–1506 (2007).
6. Kushida, K. & Kuriyama, K. Observation of the crystal-field splitting related to the Mn-3d bands in spinel-$LiMn_2O_4$ films by optical absorption. *Appl. Phys. Lett.* **77**, 4154–4156 (2000).
7. Skone, J. H., Govoni, M. & Galli, G. Self-consistent hybrid functional for condensed systems. *Phys. Rev. B* **89**, 195112 (2014).
8. Lee, S. T. *et al.* Evidence of band structure modification of $LiMn_2O_4$ upon lithium deintercalation by photoacoustic spectroscopy. *Appl. Phys. Lett.* **90**, 161912 (2007).
